# The Charge Distribution, Seebeck Coefficient, and Carrier Concentration of CuCr_0.99_Ln_0.01_S_2_ (Ln = Dy–Lu)

**DOI:** 10.3390/ma16062431

**Published:** 2023-03-18

**Authors:** Evgeniy V. Korotaev, Mikhail M. Syrokvashin, Irina Yu. Filatova, Aleksandr V. Sotnikov, Alexandr V. Kalinkin

**Affiliations:** 1 Nikolaev Institute of Inorganic Chemistry, Siberian Branch, Russian Academy of Sciences, 630090 Novosibirsk, Russia; 2 Boreskov Institute of Catalysis, Siberian Branch, Russian Academy of Sciences, 630090 Novosibirsk, Russia

**Keywords:** layered copper–chromium disulfide, XPS, lanthanides, Seebeck coefficient, Hall voltage, thermoelectricity

## Abstract

The atom oxidation states were determined using the binding energies of the core S2p-, Cu2p-, Cr2p-, and Ln3d-levels in CuCr_0.99_Ln_0.01_S_2_ (Ln = Dy–Lu) solid solutions. The charge distribution on the matrix elements (Cu, Cr, and S) remained unaffected after cationic substitution. The sulfur atoms were found to be in the S^2−^ oxidation state, the copper–Cu^+^, and the chromium–Cr^3+^. The cationic substitution of the initial CuCrS_2_-matrix occurred via the isovalent mechanism. The obtained results were compared with the electrophysical properties for CuCr_0.99_Ln_0.01_S_2_. The measured carrier concentration was from 10^17^ to 10^18^ cm^−3^. The largest Seebeck coefficient value of 157 µV/K was measured for CuCr_0.99_Yb_0.01_S_2_ at 500 K. The cationic substitution with lanthanides allowed one to enhance the Seebeck coefficient of the initial CuCrS_2_-matrix.

## 1. Introduction

The materials based on the transition metal dichalcogenides MX_2_ (M—transition metal, X—chalcogen atom) are considered promising functional materials [1,2]. Some of the MX_2_ compounds can form stable 2D single-layer structures with weak interlayer interactions. The unstable dichalcogenide MX_2_-layers can be stabilized by electropositive alkali (Li, Na, K) or transition (Cu, Ag, Au) metal atoms intercalated between the layers [3]. Thus, quasi-layered AMX_2_ (A—electropositive metal) compounds can be formed. The wide range of promising functional properties has promoted increased attention to the quasi-layered AMX_2_ dichalcogenides [4]. The functional properties of AMX_2_ can be modified. The partial cationic substitution of chalcogen or metal atoms in the MX_2_-layers is accompanied by the variation in the AMX_2_ physical and chemical properties [5]. The transformation of AMX_2_ into the thin 2D layer structures (nanosheets) allow one significantly to improve their electrical properties [6,7]. The interlayer gap of AMX_2_ can also be opened and filled with guest ions or molecules using a special synthesis approach [8]. However, cationic substitution is considered to be the most common synthesis route to enhance the functional properties of AMX_2_-based materials [9,10,11,12,13,14]. In this context, the quasi-layered copper–chromium dichalcogenide CuCrS_2_ and CuCrS_2_-based solid solutions are considered promising functional materials for their the ionic conductivity, specific electrical and magnetic properties, catalytic activity, and light-absorbing properties [10,13,15,16,17,18,19]. CuCrS_2_-based solid solutions are currently of great interest due to their promising thermoelectric properties [10,11,18,20]. The thermoelectric materials can be used for fabrication of highly efficient thermoelectric generators (TEGs) applied for wasted heat energy harvesting [21]. The high values of the Seebeck coefficient and ionic conductivity reported allow one to consider CuCrS_2_-based solid solutions as phonon–glass electron–crystal (PGEC) materials [6,14,22,23]. The Seebeck coefficient is considered to make the most significant contribution to the TEG efficiency. The materials with high values of the Seebeck coefficient can be used in thermoelectric solid-state temperature sensors [24]. Thus, increasing the Seebeck coefficient is of special interest. The Seebeck coefficient of the initial CuCrS_2_ could be improved by the cationic substitution of chromium atoms with heavier 3d- or 4f-metal atoms (dopant). For instance, the iron- or lanthanide-doped solid solutions were reported to have a promising values of the Seebeck coefficient [11,12,25,26]. The cationic substitution of Cr with heavier ions allows one to decrease the thermal conductivity due to the increase in the phonon scattering. Note that the initial CuCrS_2_-matrix can be doped in a wide range of dopant concentrations up to 40 at.% [11,27,28]. However, an increase in the dopant concentration suppresses the Seebeck coefficient of the CuCrS_2_-based solid solutions due to the metal–insulator transition (MIT) [11,27,29]. Therefore, in the present work, we report the study of the low-level concentration-doped CuCr_0.99_Ln_0.01_S_2_ (Ln = Dy–Lu) solid solutions. Note that the Seebeck coefficient of semiconductors and, namely CuCrS_2_-based solid solutions, is significantly affected by the charge carrier concentration [26,30]. In turn, the carrier concentration depends on the dopant oxidation state and determines the conductivity type. This information can be obtained using a core-level X-ray photoelectron spectroscopy (XPS) experimental technique. Here, we report the results of the comprehensive study involving the analysis of the charge distribution on the matrix elements, the charge carrier concentration, and the Seebeck coefficient value for CuCr_0.99_Ln_0.01_S_2_ (Ln = Dy–Lu).

## 2. Experiment

**Figure 1:**
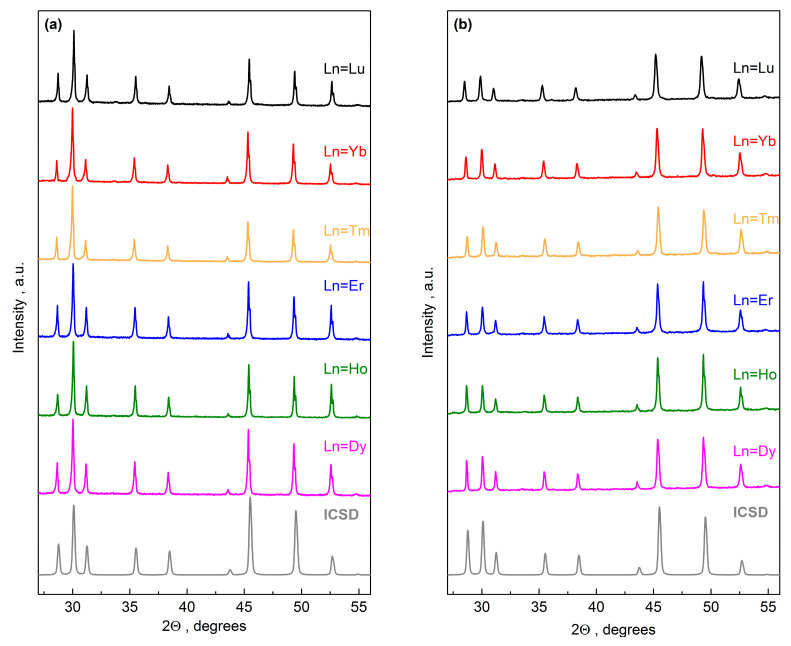
The XRD patterns for CuCr_0.99_Ln_0.01_S_2_ (Ln = Dy–Lu): powder (**a**) and ceramic (**b**) samples.

Powder samples of the CuCr_0.99_Ln_0.01_S_2_ (Ln = Dy–Lu) solid solutions were obtained using solid-state sulfidization of the commercial metal oxides CuO, Cr_2_O_3_ (MilliporeSigma, Burlington, NZ, USA), and Ln_2_O_3_ (Ln = Dy … Lu) (Novosibirsk Rare Earth Metal Plant, Russia) with a purity of 99.99%. The stoichiometric mixture of the initial oxides in a glassy carbon crucible was placed in a horizontal high temperature quartz tube furnace. The sulfidation procedure was carried out in an argon flow at 1050–1100 °C with the gaseous products of ammonium rhodanide (NH_4_SCN) decomposition [25,31]. The heating rate was 60 °C/min. After the sulfidation procedure, the sample was kept in the furnace and cooled to room temperature in an argon flow for 30 min. Then, the sample was ground. The synthesis conditions are listed in Table 1. The completeness of the sulfidation was controlled by the powder X-ray diffraction (XRD) on a Bruker D8 Advance diffractometer using CuKα-radiation (λ = 1.5418 Å). The XRD patterns after the final sulfidation stage are plotted in Figure 1a. The synthesized samples were composed of a single phase corresponding to the rhombohedral *R3m* space group [32]. The diffraction peak positions were in good agreement with the XRD data of the Inorganic Crystal Structure Database (ICSD) for the initial CuCrS_2_-matrix (ICSD ID 100594).

The X-ray photoelectron (XPS) study of CuCr_0.99_Ln_0.01_S_2_ (Ln = Dy–Er) was carried out using a SPECS spectrometer with a PHOIBOS-150 hemispherical electron energy analyzer. The XPS measurements of CuCr_0.99_Ln_0.01_S_2_ (Ln = Tm–Lu) were carried out using an ESCALAB 220i spectrometer. The XPS lines (Cu2p, Dy3d, S2p) were measured using a nonmonochromatic AlKα-radiation source (hν = 1486.6 eV). The Cr2p-lines were measured using a nonmonochromatic MgKα-radiation source (hν = 1253.6 eV). The samples were fixed on a substrate using double-sided adhesive conductive carbon tape. The spectrometer energy scale was calibrated using the reference binding energy (BE) values for the metallic gold Au4f_7/2_ (84.0 eV) and copper Cu2p_3/2_ (932.6 eV). The measured BE values were corrected using the carbon C1s-line (284.8 eV) of an adventitious carbon in the near-surface layers of the samples [33,34]. The XPS lines were processed using CasaXPS 2.3.15 software. The measurement accuracy of the BE was 0.2 eV.

The powder samples were used to prepare the cylindrically compressed ceramic samples using a laboratory-made setup. The ceramic samples were compressed in a vacuum (5 × 10^−5^ Torr) using the hot-pressing consolidation technique under a uniaxial pressure of 70 MPa at 923 K for 1.5 h [35]. The XRD patterns for the obtained ceramic samples indicated the preservation of the crystal structure of the initial powders (Figure 1b).

The Seebeck coefficient was measured in a rarefied helium atmosphere with the residual pressure of 5 Torr [11]. The ceramic sample was placed between two contact copper pads with a temperature gradient of 5 K controlled by a Thermodat-13K5 temperature controller. The thermoelectric power arising from the sample was measured using a 6½ Keysight 34465A multimeter. The measurement accuracy of the thermopower was 5%.

The Hall voltage measurements were carried out using the Van der Pauw technique at room temperature using a laboratory-made setup. The DC magnetic field of 1T was applied perpendicular to the current and sample plane. The current of 10 mA flowing through the sample was controlled with an LM334 stabilizer. The Hall voltage was measured using a 6½ Keysight 34461A multimeter. During the measurements the direction of the current and magnetic field polarity were reversed. Then, the current and potential probes were swapped. The Hall voltage value was the result of eight independent measurements. The Hall voltage polarity was calibrated according to the reference sample of a p-type silicon wafer.

## 3. Results and Discussion

**Figure 2:**
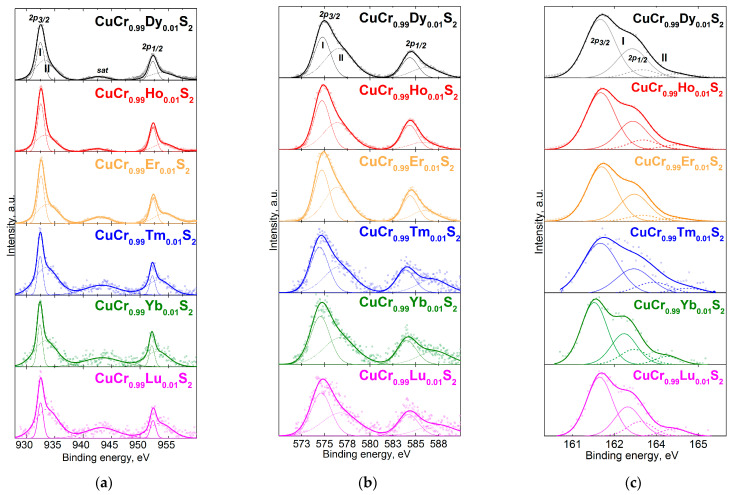
The XPS lines for CuCr_0.99_Ln_0.01_S_2_ (Ln = Dy, Ho, Er, Tm, Yb, Lu): (**a**) Cu2p-region; (**b**) Cr2p-region; (**c**) S2p-region. Note: I and II corresponded to the different atom species.

The XPS Cu2p-region for the CuCr_0.99_Ln_0.01_S_2_ (Ln = Dy–Lu) solid solutions are plotted in Figure 2a. The binding energy (BE) values for the measured Cu2p-, Cr2-, Dy3d-, and S2p-lines are listed in Table 2. The Cu2p-region exhibited two intense lines originating from the spin-orbit splitting of the Cu2p_3/2_- and Cu2p_1/2_-levels. The intense line (denoted as I in Figure 2a) with the BE of 932.3–932.6 eV corresponded to the Cu^+^ state, as reported previously [26]. Note that the satellite lines (denoted as “sat” in Figure 2a) at ~941 eV indicated the presence of the Cu^2+^ state [36,37]. The high energy shoulder (II in Figure 2) of both the Cu2p_3/2_- and Cu2p_1/2_-lines (~933.3 and 953.8 eV, respectively) was observed due to the Cu^2+^ in the defective near-surface layers. This fact was in good agreement with the previously reported data for CuCrS_2_-based solid solutions [12,26,28,33]. Thus, one can conclude that the oxidation state of the copper atoms in the composition of CuCr_0.99_Ln_0.01_S_2_ (Ln = Dy–Lu) was Cu^+^ as was reported for the lanthanide series from lanthanum to terbium in CuCr_0.99_Ln_0.01_S_2_ solid solutions [12,26].

Figure 2b plots the Cr2p-region for the solid solutions studied. The Cr2p-region exhibited two intense peaks originating due to the spin-orbit coupling of the core Cr2p_1/2_- and Cr2p_3/2_-levels with a BE of ~575 and ~584 eV, respectively. The low-energy component of Cr2p_3/2_ with the BE of 574.5–574.8 eV was attributed to the Cr^3+^ (denoted as I in Figure 2b). The high-energy component with the BE of 576.4–576.8 eV was attributed to the chromium atoms in the oxygen-containing compounds in the near-surface layers. Note that the presence of the corresponding components in the Cr2p-region was in accordance with the data concerning the CuCrS_2_-based solid solutions [12,26,33]. Based on the Cr2p_3/2_-line BE value, the chromium atoms in the composition of the CuCr_0.99_Ln_0.01_S_2_ were considered as Cr^3+^.

The S2p-region exhibited two sets of lines originating from the different species of sulfur atoms (denoted as I and II in Figure 2c). The low-energy set with the BE of 161.1–161.6 eV (Table 2) referred to the S^2−^ state and related to the sulfur atoms in the CuCr_0.99_Ln_0.01_S_2_. The high-energy set with the BE of 162.9–163.0 eV originated from the sulfur atoms of the polysulfide anions and the elemental sulfur in the defective near-surface layers [38,39]. Note that the presence of the additional surface sulfur species is typical for CuCrS_2_-based compounds [26,28,33]. Thus, the oxidation state of the sulfur atoms in CuCr_0.99_Ln_0.01_S_2_ was considered as S^2−^.

**Figure 3:**
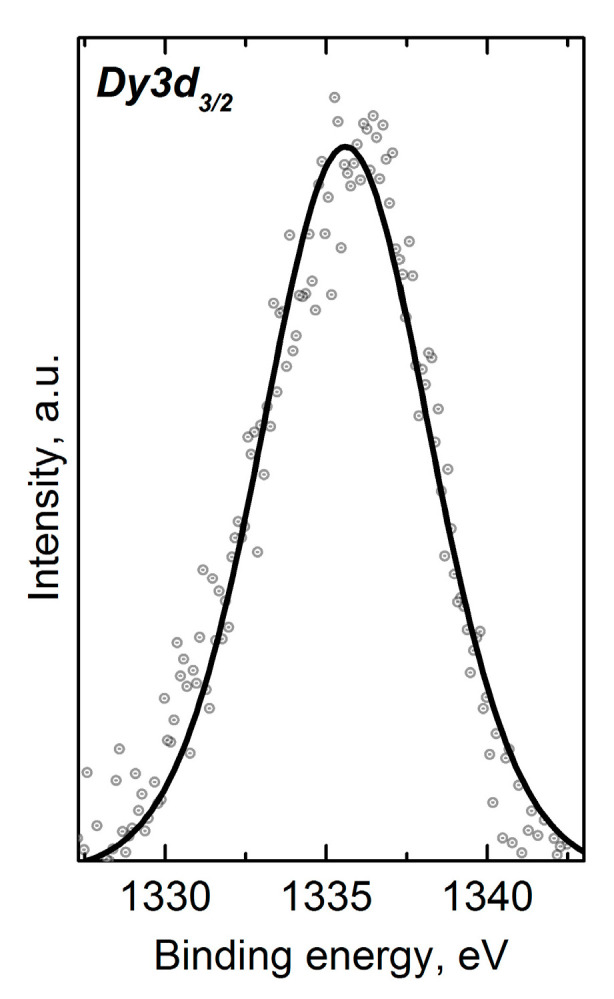
XPS Dy3d_3/2_-region for CuCr_0.99_Dy_0.01_S_2_.

The oxidation state of the lanthanides is of special interest due to the fact that the contribution of the Ln4*f*-level is assumed to affect the electronic structure reconstruction and thereby the physical properties of CuCr_0.99_Ln_0.01_S_2_ [12]. However, the Ln3d core-level BE for lanthanides from Ho to Lu were greater than the excitation energy that could be reached using a laboratory XPS spectrometer with a twin Al/Mg anode X-ray source. However, the Ln4d-lines could not be measured due to the low concentration of lanthanides. In this regard, the core-level energy region was measured only for the Dy-doped solid solution (Figure 3). The measured BE value of 1335.6 eV for the Dy3d_3/2_ was typical for Dy^3+^ compounds [40,41]. Note that the obtained results were in accordance with the previously reported data concerning the magnetic properties of CuCr_0.99_Ln_0.01_S_2_ (Ln = La–Lu) solid solutions [32]. The correlation between the experimental and the theoretical dependencies of the magnetic moment allowed one to consider the lanthanide oxidation state as Ln^3+^. This fact indicated the isovalent Cr^3+^→Ln^3+^ cationic substitution mechanism in CuCr_0.99_Ln_0.01_S_2_ solid solutions.

**Figure 4:**
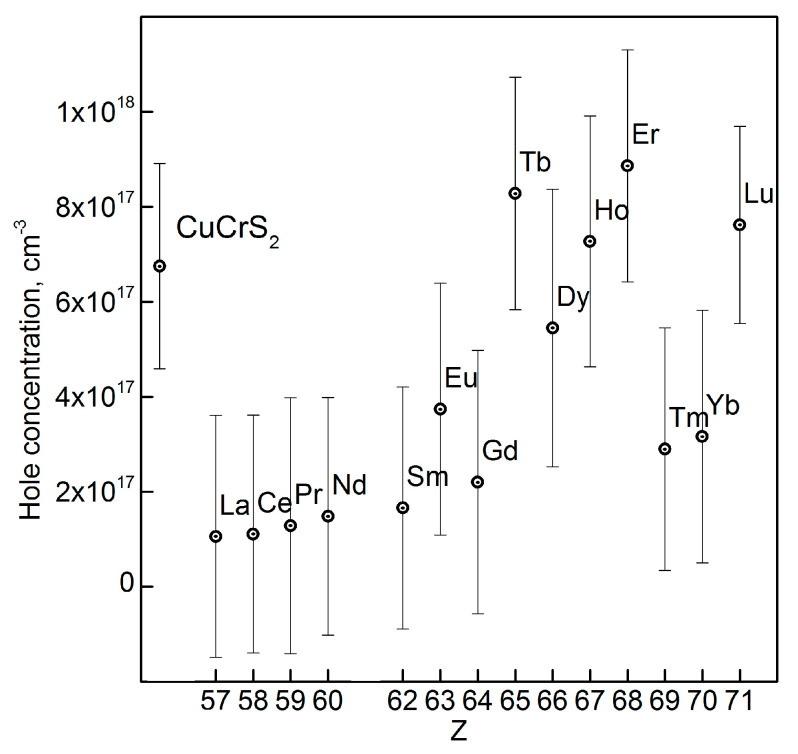
The hole concentration for the CuCr_0.99_Ln_0.01_S_2_ (Ln = La–Lu).

The absence of the significant chemical shifts of the core Cu2p-, Cr2p-, and S2p-lines for the CuCr_0.99_Ln_0.01_S_2_ solid solutions indicated the preservation of the electron density on the matrix elements during the cationic substitution of Cr^3+^ with Ln^3+^ ions. Note that the lanthanide atom type variation does not significantly affect the oxidation state of the matrix elements (Cu, Cr, and S) [12,26,32]. Thus, the lanthanide atoms in the composition of solid solutions should exhibit neither donor nor acceptor behavior with respect to the initial matrix. The observed trend was characteristic for the lanthanide row from La to Lu [12,26,32]. In this context, the carrier concentration for the initial CuCrS_2_-matrix and CuCr_0.99_Ln_0.01_S_2_ solid solutions was studied using the Hall voltage measurements. The measured Hall voltage values were used to calculate the carrier concentration in terms of the strong magnetic field approximation. A positive sign of the Hall voltage indicated the p-type of the conductivity for CuCr_0.99_Ln_0.01_S_2_. The charge carrier concentration value as a function of the atomic number Z for CuCr_0.99_Ln_0.01_S_2_ (Ln = La–Lu) is depicted in Figure 4.

**Figure 5:**
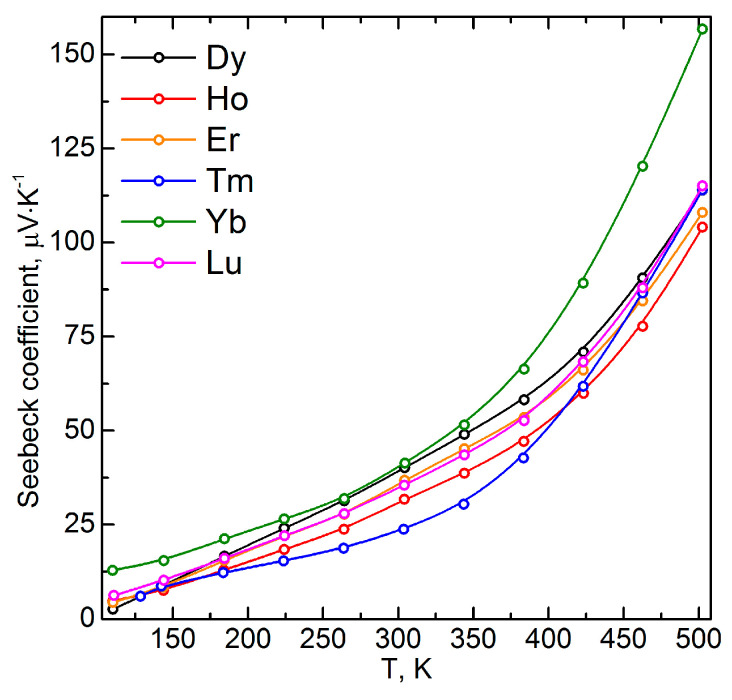
The Seebeck coefficient for the CuCr_0.99_Ln_0.01_S_2_ (Ln = Dy–Lu).

The lanthanide atom type affected the charge carrier concentration value. Taking into consideration the isovalent mechanism of the cationic substitution, one can conclude that the observed variation in the charge carrier concentration was due to the electronic structure reconfiguration, the redistribution of the partial density of states (DOS) in the conduction, and the valence bands. The cationic substitution led to the emergence of the additional lanthanide f-states in the Fermi-level region [12,26]. For instance, in case of lanthanides from La to Gd, a charge carrier concentration decrease was observed, compared to the initial CuCrS_2_-matix. The solid solutions with Tb, Dy, Ho, Er, and Lu exhibited the carrier concentrations comparable to those for the CuCrS_2_-matix. Thus, the carrier concentration was determined by the 4f-orbital population. The obtained values for the Yb- and Tm-doped solid solutions could be due to the deeper localization of the f-states in the valence band compared to the other lanthanides. The positive sign of the Seebeck coefficient also indicated the p-type of the conductivity (Figure 5). The largest Seebeck coefficient value of 157 µV/K was observed for the CuCr_0.99_Yb_0.01_S_2_ at 500 K. The obtained value was 1.5 times greater in comparison with those for the initial matrix (105 µV/K) [26]. Thus, based on the data reported previously, one can conclude that the cationic substitution of the initial CuCrS_2_-matrix with the entire lanthanide row from La to Lu enhanced the Seebeck coefficient [12,25,26]. The most promising value of 325 µV/K was reported previously for the La-doped solid solution at 500 K [25]. Note that the observed Seebeck coefficient increase for the La- and Yb-doped solid solutions correlated with the charge carrier concentration decrease. The enhancement of the thermoelectric properties was due to the electronic structure reconstruction near the Fermi-level region (i.e., the valence band top and conduction band bottom) after the cationic substitution of chromium atoms in the CuCrS_2_-matrix with lanthanide ions [12,42].

## 4. Conclusions

It was found that the cationic substitution of the CuCrS_2_-matix with lanthanides (Dy–Lu) occurred via the isovalent mechanism. The oxidation state of the copper atoms in CuCr_0.99_Ln_0.01_S_2_ (Ln= Dy–Lu) was found to be in the monovalent Cu^+^ oxidation state. The sulfur atoms in CuCr_0.99_Ln_0.01_S_2_ were found to be in the S^2-^ oxidation state. The chromium and lanthanide atoms were found to be in the trivalent Cr^3+^ and Ln^3+^ oxidation state, respectively. The obtained data concerning the charge distribution analysis were compared with the results of the electrophysical measurements. It was found that the dopant atoms in CuCr_0.99_Ln_0.01_S_2_ solid solutions did not exhibit either donor or acceptor behavior due to the isovalent cationic substitution mechanism. The observed changes of both the carrier concentration and the Seebeck coefficient were related to the redistribution of the CuCr_0.99_Ln_0.01_S_2_ electronic structure during the cationic substitution. The largest Seebeck coefficient value of 157 µV/K was observed for the CuCr_0.99_Yb_0.01_S_2_ at 500 K. The measured value was 1.5 times greater compared to the initial CuCrS_2_ (105 µV/K). It was found that the cationic substitution with the lanthanide atoms allowed one to improve the electrophysical properties of the initial CuCrS_2_-matrix. The observed trend was characteristic for the entire lanthanide row from La to Lu. The most promising value of 325 µV/K was reported previously for the La-doped solid solution. The observed Seebeck coefficient increase for the La- and Yb-doped solid solutions correlated with the carrier concentration decrease. The observed enhancement of the electrophysical properties of CuCr_0.99_Ln_0.01_S_2_ (Ln = La–Lu) was assumed to occur due to the contributions of the lanthanide f-states in the electronic structure after the substitution of the chromium atoms with lanthanide ions in the initial CuCrS_2_-matrix.

## Figures and Tables

**Table 1:** Synthesis conditions for CuCr_0.99_Ln_0.01_S_2_ (Ln = Dy–Lu).

	Temperature (°C)	Duration (h)
CuCr_0.99_Dy_0.01_S_2_	300 550 700 950 * 1050 * 1050 *	0.25 0.30 2 4 4 3
CuCr_0.99_Ho_0.01_S_2_	300 550 700 950 * 1050 * 1050 *	0.25 0.30 2.6 2.5 4 2.5
CuCr_0.99_Er_0.01_S_2_	300 550 700 950 * 1100 * 1100 *	0.25 0.30 1 4.6 2.25 4
CuCr_0.99_Tm_0.01_S_2_	300 550 700 950 * 1000 * 1100 * 1100 * 1100 *	0.25 0.30 3 0.6 2.5 3.15 =3.15 3
CuCr_0.99_Yb_0.01_S_2_	300 550 700 950 * 1100 * 1100 * 1100 *	0.25 0.30 3 2.75 2 2.5 3.25
CuCr_0.99_Lu_0.01_S_2_	300 550 700 * 950 * 1000 * 1000 * 1050 *	0.25 0.30 3.15 4.30 4 4.3 4.5

* the sample was ground after the sulfidation procedure.

**Table 2:** Binding energy values for the Cu2p-, Cr2p-, S2p-, and Dy3d-lines.

	Cu2p_3/2,1/2_	Cr2p_3/2,1/2_	S2p_3/2_	Dy3d_3/2_
CuCr_0.99_Dy_0.01_S_2_	932.5 933.3	574.8 576.6	161.5 163.0	1335.6
CuCr_0.99_Ho_0.01_S_2_	932.6 933.3	574.7 576.4	161.5 163.0	–
CuCr_0.99_Er_0.01_S_2_	932.3 933.7	574.7 576.4	161.6 163.0	–
CuCr_0.99_Tm_0.01_S_2_	932.5 933.5	574.5 576.6	161.1 162.8	–
CuCr_0.99_Yb_0.01_S_2_	932.4 933.4	574.6 576.7	161.3 162.7	–
CuCr_0.99_Lu_0.01_S_2_	932.5 933.6	574.7 576.8	161.5 162.9	–

## Data Availability

Not applicable.
